# Lost in translation: a focus group study of parents’ and adolescents’ interpretations of underage drinking and parental supply

**DOI:** 10.1186/s12889-016-3218-3

**Published:** 2016-07-13

**Authors:** Sandra C. Jones, Kelly Andrews, Nina Berry

**Affiliations:** Centre for Health and Social Research (CHaSR), Australian Catholic University, Level 5, 215 Spring St., Melbourne, VIC 3000 Australia

**Keywords:** Alcohol, Underage drinking, Social norms, Social marketing, Focus groups, Theory of planned behaviour

## Abstract

**Background:**

Reductions in underage drinking will only come about from changes in the social and cultural environment. Despite decades of messages discouraging parental supply, parents perceive social norms supportive of allowing children to consume alcohol in ‘safe’ environments.

**Methods:**

Twelve focus groups conducted in a regional community in NSW, Australia; four with parents of teenagers (*n* = 27; 70 % female) and eight with adolescents (*n* = 47; 55 % female). Participants were recruited using local media. Groups explored knowledge and attitudes and around alcohol consumption by, and parental supply of alcohol to, underage teenagers; and discussed materials from previous campaigns targeting adolescents and parents.

**Results:**

Parents and adolescents perceived teen drinking to be a common behaviour within the community, but applied moral judgements to these behaviours. Younger adolescents expressed more negative views of teen drinkers and parents who supply alcohol than older adolescents. Adolescents and parents perceived those who ‘provide alcohol’ (other families) as bad parents, and those who ‘teach responsible drinking’ (themselves) as good people. Both groups expressed a preference for high-fear, victim-blaming messages that targeted ‘those people’ whose behaviours are problematic.

**Conclusions:**

In developing and testing interventions to address underage drinking, it is essential to ensure the target audience perceive themselves to be the target audience. If we do not have a shared understanding of underage ‘drinking’ and parental ‘provision’, such messages will continue to be perceived by parents who are trying to do the ‘right’ thing as targeting a different behaviour and tacitly supporting their decision to provide their children with alcohol.

## Background

In Australia the national guidelines state that for those aged less than 18 years of age, not drinking is the safest option [1]. Adolescent drinking is associated with increased risk of accidents and injury, including vehicle accidents [2], suicide and violence [3, 4]. Regular alcohol consumption or binge drinking during adolescence also associated with a range of negative health and social outcomes including physical and mental health problems, risky sexual behaviour, poor school performance and anti-social behaviour; and long-term health effects such as alcohol dependence and poor health outcomes in early and middle adulthood [2, 5, 6]. However, in 2011, 51 % of Australian children aged 12–17 reported consuming alcohol in the previous 12 months; 17 % in the last week [7].

The influences on adolescent drinking behaviour are complex. There is strong evidence for the role of alcohol advertising and marketing [8, 9], personality factors [10, 11] and the drinking behaviours and attitudes of peers [12–15], and siblings [16]. However, a growing body of evidence indicates that parents exert significant influence over adolescents’ underage drinking behaviour; children of parents permit or accept underage drinking are more likely to consume alcohol in adolescence than those whose parents apply prohibitions or strict rules and emphasise the negative effects of alcohol [12, 17, 18].

There are considerable variations in reports of parental supply of alcohol between countries, and between parents and adolescents within countries [19]. However, it is clear that parents are a common source of alcohol for (particularly younger) adolescents; with this finding being replicated in a range of countries including the United States [20, 21], Sweden [22], Ireland [23] and Australia [24]. In the 2011 Australian secondary school survey, 34.9 % of 12-to-15-year-olds and 31.3 % of 16-to-17-year-olds report that their last alcoholic drink was provided to them by their parents [7]; and in a survey of 530 secondary students in New South Wales, 40.7 % of drinkers reported receiving alcohol from their parents in the last month, with younger respondents were more likely to report that their parents were their *main* source of alcohol [25].

An analysis of data from the 2007 Australian National Drug Strategy Household Survey found that adolescent drinkers who recalled receiving their first drink from their parents had lower rates of risky drinking than those who recalled receiving their first drink from another source [26]; and the analysis of youth data from the US National Survey on Drug Use and Health showed that those who obtained their last drink from a parent or family member drank less frequently and at lower levels than those who obtained their alcohol from an unrelated adult or purchased it themselves [27]. Conversely, studies in Sweden [28], Australia [25], and concurrently in the US and Australia [29] have shown no evidence that parental supply leads to more ‘responsible’ drinking patterns, rather that adolescents were more likely to engage in harmful drinking behaviours if their parents provided them with alcohol.

Determining the exact nature of this relationship is complicated by the combination of source of supply (parent or other), location of drinking, presence or absence of supervision, and other contextual or situational differences. For example a cross-sectional telephone survey of 6245 US adolescents found that adolescents who were with their parents last time they drank alcohol reported less frequent and more moderate drinking, whereas those whose parents or friends’ parents^1^ had provided them with alcohol at a party reported more frequent and more hazardous drinking [21]. Similarly, an Australian survey of 530 secondary students found that those who were provided alcohol by their parents for consumption without (their own) parental supervision were more likely to be risky drinkers [25]. Finally, data from the 2011 Australian national school survey reported that current drinkers drank less alcohol per week if they obtained their alcohol from their parents and drank less if they consumed the alcohol at home; however 16–17-year-olds who drank at a party consumed significantly less if friends supplied the alcohol than if parents or someone else provided it [7].

It is well-documented that adolescents perceive strong descriptive norms encouraging drinking and weak injunctive norms discouraging drinking [30–33]. There is increasing evidence that parents perceive similar norms in relation to the provision of alcohol to adolescents and that perceived norms surrounding the drinking behaviours condoned by ‘other parents’ may influence parental attitudes [34]. However, there are also a small number of studies that suggest that adults perceive that their own views are comparatively conservative and that the broader community is more accepting of underage drinking than they are themselves [35, 36].

Thus, it is clear that strategies to address the problem of underage drinking need to reach beyond targeting young people themselves and begin to address the attitudes and values held by parents and community members [37]. Reductions in underage drinking will only come about from changes in the social and cultural environment in which our young people are learning about the role of alcohol in their lives. In recognition of the important role of parents in discouraging (or facilitating) alcohol consumption, several interventions in the US [38, 39], and Europe [40] have included components targeting teenagers and their parents, individually and concurrently.

This paper reports a qualitative study of adolescents and parents in a regional town New South Wales (Australia), a jurisdiction where it is not unlawful for children or adolescents to *consume* alcohol. Rather, it is unlawful for a person who is less than 18 years of age to purchase alcohol or to consume alcohol on licensed premises or in public places; or to sell alcohol to a person under the age of 18, or to supply them with alcohol in the absence of parental consent to do so. It is not unlawful for parents to provide alcohol to their own children for consumption in a private home or for others to provide alcohol to children for consumption on private premises (not including licensed premises), if parental consent is obtained.

The study explored knowledge, attitudes and experiences of the supply of alcohol to minor (not yet 18) children by their parents. The aim of the study was to investigate adolescents’ and parents’ perceptions of these behaviours, and their perceptions (and perceived personal relevance) of previous social marketing campaigns.

The study was informed by the Theory of Planned Behaviour (TPB), which posits that attitudes, subjective norms and perceived behavioural control predict behavioural intention [41, 42]. The TPB has been used extensively to explain drinking and drinking intentions among young adults, such as college students [43–47], more recently in studies among adolescents [48, 49] and has potential for understanding parental supply of alcohol [19]. For example, a study of 247 secondary school pupils (mean age 16.6 years) across a range of health-related behaviours (including drinking alcohol) found TPB, together with past behaviour, explained 62 % of the variance in health-risk intentions, and 51 % of the variance in health-protective intentions [50].

## Methods

The study was conducted in a local government area (LGA) in New South Wales, Australia, that is 120 km from the state’s capital city. The LGA includes a town with a population of approximately 20,000 and has a Socio Economic Index for Area (SEIFA; general level of socio-economic (dis)advantage of people living in the area) value of 1055, which is above the mean.^2^ The town has one publically funded high school and its own weekly newspaper, and the median weekly household income is AUD1,099 AUD.

Focus group participants were recruited by advertisements placed in the local newspaper, community newsletters and on social media platforms; and posters displayed in community spaces including libraries, doctors’ surgeries and shopping centres. Twelve focus groups were conducted in venues such as libraries, the local high school and neighbourhood centre: four with parents of teenagers (*n* = 27) and eight with adolescents (three groups of 12–14-year-olds, three groups of 15–17-year olds, and two mixed groups; total *n* = 47). One parent group and one group of 12–14-year-olds were conducted concurrently and consisted predominantly of members of the same families; it is possible that some of the other adolescent participants may have been related to adult participants but did not disclose this.

Focus groups generally ran for one hour and consisted of 6–10 participants who were offered retail vouchers in return for their time ($50 for adults and $30 for adolescents). All discussions were audio-recorded and later transcribed in full. Recruitment methods, focus group discussion guides and all data collection methods were approved by the University of Wollongong’s Human Research Ethics Committee. Adult participants provided written consent, and adolescent participants provided both written assent and written parental consent; this included consent to publish the de-identified findings. The Consolidated Criteria for Reporting Qualitative Findings (COREQ) [51] was used to provide a systematic framework for the design, analysis and reporting of this study. A comprehensive checklist against the 32-item COREQ as well as all other transcripts and data files can be obtained from the first author upon request.

Basic demographics of participants were recorded at the commencement of each focus group using a self-completion survey (see Table 1). The mean age of adolescent participants was 14.8 years (range 12–17 years) and of parents was 46.7 years (range 37–62 years); 55.3 % of adolescents and 70.4 % of parents were female. The majority of adolescents and parents were born in Australia and spoke English at home.

**Table 1:** Participant demographics

	Adolescents (*n* = 47)		Parents (*n* = 27)	
	%	n	%	n
Gender				
Male	44.7 %	21	29.6 %	8
Female	55.3 %	26	70.4 %	19
Country of birth				
Australia	89.4 %	42	81.5 %	22
Other	10.6 %	5	18.5 %	5
Language spoken at home				
English	93.6 %	44	92.6 %	25
Other	6.3 %	3	7.4 %	2
Religion				
Catholic	31.9 %	15	18.5 %	5
Anglican	19.1 %	9	14.8 %	4
Other religion	4.2 %	2	22.6 %	6
No religion	44.7 %	21	44.4 %	12

Focus group discussions then followed a thematic discussion guide which explored knowledge and attitudes and around alcohol consumption by, and parental supply of alcohol to, underage teenagers. Following this, the groups were shown examples of print materials from social marketing campaigns to reduce underage drinking and participants engaged in a discussion regarding their opinions on the various campaigns.

For adolescent groups, in addition to discussion guides, facilitators used activities to stimulate discussion. Groups comprising 12–14 year olds were given picture and photo sorting activities; and groups comprising 15–17 year olds adjectival word sorting activities. These activities focused on sorting and then discussing impressions of ‘drinkers’ and ‘non-drinkers’ and of parents who ‘do’ and ‘do not’ provide alcohol to their teenage children.

The first and second authors, both experienced qualitative researchers, independently examined and manually coded the data. Both coders commenced by grouping quotes under the key headings of the Discussion Guide, itself designed to elicit responses corresponding to the constructs of the TPB (attitudes towards underage drinking and parental supply, subjective norms and perceived behavioural control), and then coded for specific issues and themes. The two coders met to consider these key groupings and to resolve any differences in interpretation. The third author reviewed the draft findings, then read the verbatim transcripts to confirm the clarity and accuracy of interpretation.

## Results

### Attitudes towards underage drinking

In general, the responses of the younger teenagers (12–14yo) showed strong health and moral judgements (injunctive norms) being applied to teenage drinking. This group viewed drinkers as simply ‘bad’ and non-drinkers as simply ‘good’.

*I reckon they’re probably all nice people but if you get drunk you can’t really keep being nice because you just get all aggro and stuff*

*(A1 – younger group)*

*I don’t think this one drinks at all because she’s like a model so that means she’s successful and she’s got a great smile.*

*(A3 – younger group)*

The older teenagers found it more difficult to apply simple negative and positive adjectives to drinkers and non-drinkers. This group expressed more open and accepting views towards drinking. They acknowledged that drinkers were often more ‘annoying’, ‘silly’ and ‘aggressive’ whilst emphasising that drinking could be associated with positive (or neutral) characteristics and outcomes.

*People when they’re drunk lose reality a bit so they’re not stressed or self conscious because they’re just ‘yeah, whatever’*

(A6 – older group)

There was a general consensus among the parent participants that local teenagers (especially those aged 16–17yo) are drinking, *“90 percent at least” – “I’d say 85”,* but that their own teenagers are not; *“Having already had a child grow up and listening to the stories over the years – he [my son] didn’t but other kids did*” (P1). There was also a recognition that the child’s peer group was an important influence on their drinking behaviour,

*I think there are lots that aren’t doing it – that aren’t drinking…*

*It depends on your peer group I think* (P1)*.*

Parent groups also expressed the view that, whilst peer pressure and other environmental factors influence adolescent drinking behaviour, parents themselves are also a significant influence either by role modelling their own behaviour or by the degree of parental rules and boundaries. In this context there was a clear distinction made between parents who drink ‘responsibly’ and those who drink to excess.

*They’re maybe big drinkers themselves. I just think it’s that thing, if it’s in your environment, where it’s the norm, where every night at dinner there’s lots of alcohol being consumed and every BBQ adults are getting tiddly it’s an environment where children are used to it being normal, whereas we – they see us just have one glass of wine, it’s very different to seeing a parent getting drunk* (P1)

*They’re allowing parties to go on at their place at 16 and knowing there are kids out there drinking in their garden and things like that…Some parents just think it’s normal because it’s probably what they did* (P4)

### Attitudes towards parental supply of alcohol

At a theoretical level the teenagers, and particularly the younger respondents, expressed the view that ‘good’ parents don’t let their children drink. For example, the 12–14-year-olds allocated the photos of the ‘nice’ and ‘happy’ parents to the ‘do not provide alcohol’ pile and the ‘angry’ and ‘unhappy’ parents to the ‘do provide’ alcohol pile.

[do not provide] …. *anniversary or romantic so I don’t think that they would let their kids drink because they look like nice people* (A1)

[do not provide] *If you look at the ‘don’t’ pile, a lot of them look angry or less happy* (A2)

[do provide] *I think they look like the kind of relaxed person that doesn’t care what their kids do* (A1)

[do provide] *These people look like parents who just leave their children at home and go out to find drugs* (A2)

Perceptions among the older teenagers were more varied and more nuanced. Some thought the supply of alcohol to adolescent children was ‘bad’ parenting (as was the case with the younger group), whereas others saw it as harm minimisation. These respondents also expressed mixed views of parents who do not provide alcohol. Some viewed this as lack of trust or respect for their children whereas others viewed parental prohibition more positively.

[do not provide] *Offensive - A few friends at school and stuff are allowed to drink and then if you tell the parent that you want to drink and they say ‘oh no, you’re too young’ you feel insulted your parents are saying you’re too young* (A4)

[do not provide] I: *If we put awesome over there then why is exciting and amazing on this…*

P: *Parents can still be cool if they don’t let you go out*

P: *They just might be against it* (A4)

[do provide] *Neglectful….If the person doesn’t care about their kid they’re neglecting their kid. I think they don’t care if the kids drinking* (A4)

[do provide] *They know their kid is going to go out and drink whether they say they can or not so they may as well know what they’re doing, know what they’re drinking rather than ‘behind my back they’re going to do this, I’d like to know at least’* (A5)

Among parents, there was a clear perception that supplying alcohol to teenagers was inappropriate. Related to this was the clear perception that ‘other parents’ *do* supply alcohol to their teenage children. While some perceived this as driven by misperceptions about harm minimisation, the majority attributed it to poor parenting.

*But I think most parents, if they are letting their kids [drink], maybe there is that thinking that it is a better way to approach it. I don’t think most parents would choose to give their children alcohol knowing it has a negative effect*.

*Unless they have a problem themselves* (P1)

*Do you know what? It’s because they just don’t want to keep hounding the children. They’ve just given up* (P2)

*There are a lot of different socio economic areas in Kxxx and that’s where sometimes you tend to find – different families…the kids are often left to their own devices a bit more* (P3)

### Experiences of parental supply of alcohol

The responses from teenagers suggested their understanding of the notion of ‘supply’ differed from the strict legal definition (“supply” includes sell and distribute, and also includes agreeing to supply, or offering to supply, or keeping or having in possession for supply, or sending, forwarding, delivering or receiving for supply, or authorizing, directing, causing, suffering, permitting or attempting any of those acts or things” [52]. In fact, their responses suggested that ‘supply alcohol’ may have a very specific meaning to this group.

At a personal level, the teenagers in the 12–14 year old groups reported that their parents don’t let them drink. A minority described absolute prohibition, generally related to their parents’ professional or personal experience of people with alcohol problems. However, the majority of them described their parents allowing them or their siblings to ‘taste’ or ‘sip’ alcohol. Some described being allowed tastes or sips of alcohol under direct parental supervision as being ‘taught how to drink’, even expressing the view that these experiences do not count as ‘drinking’. None expressed the view that this practice constituted ‘supplying alcohol’ to them.

*My mum knows how much drinking messes you up and she would never ever let me drink because I’m in a family of 8 kids* (A2)

*My mum thinks a glass of wine at a family dinner for me, mixed with something else is ok but only at a family dinner which we don’t have often* (A2)

*But it was only a tiny bit. They wouldn’t let me have a full on drink, like a full on beer but my sister, she’s 16, she likes Cruisers already* (A1)

A similarly nuanced view of what constitutes ‘drinking’ (and thus ‘supply’) was evident in the older (15–17yo) groups, although in this case distinction was made between ‘responsible’ or light drinking and ‘risky’ drinking. The majority believed that their parents would (or do) allow them to drink (responsibly) but would not allow them to drink where the goal or result of the drinking was intoxication. Some expressed the view that being allowed to drink (responsibly) is a developmental or trust issue and others that it is a concession to the inevitability of underage drinking.

*…one beer, my dad would accept, not too much more* (A6)

*I know a friend whose mum is against it but won’t stop it from happening. She’s ‘don’t do it, don’t do it but once it happens she’s like huh’ [i.e. dismissive]* (A4)

There was also a clear perception that parental provision of alcohol, within their own families, was situation-dependent. Respondents described parents making decisions based on a considered risk assessment rather than applying a single rule to every situation.

*It depends who you’re with, where you’re going and how you’re getting home*

*How you’re getting home*

*I reckon where you’re going for starters*

*Yeah…going to a party with just – locally – with a group*

*How many people too, that’s a factor*

*It’s a party that your friend at school is holding and you know everyone there – but if you go to a party at Axxx and don’t know anyone, it’s just an open house, then they’re obviously going to get –*

*Even if you can just catch a train home, they’ll be like ‘yeah – no’*

(A4)

*It depends, on what….if it’s at a party that one of your mates is having, that all the people you know then it might be all right. But if it’s…stupid people… places, like Wxxx and stuff….* (A5)

The majority of parents were adamant that they did not, and would not, supply alcohol to their underage teenagers. For many this was associated with being part of social networks that held similar attitudes. This was particularly the case when discussing younger teenagers (i.e., 14 and 15 year olds).

*No I don’t know anyone*

*I don’t, but I don’t think that means it doesn’t happen*

*I just think I probably have similar philosophies as people who are close to me*

*Like minded*

(P1)

*I don’t know any that let under 18s drink*

*No*

*Not a single one*

(P3)

It became clear as the discussion progressed that the nuanced perception of what constitutes ‘drinking’ and ‘supplying alcohol’ expressed in the adolescent groups was shared by the parents. That is, while the majority were adamant that they would not supply alcohol to their underage teenagers (and that those who did were either unwise or neglectful), many referred to giving their children ‘tastes’ of alcohol or ‘teaching’ them to drink responsibly by introducing them to alcohol in a ‘safe’ environment.

*…but I’ve let X try my wine probably since he was about 11 – a sip. Because I’d rather him know what a good wine tastes like and I say to him what can you taste and smell, think about it like proper wine tasting. Because I’d rather him have an appreciation for a good wine ….* (P2)

*…but I would let my child at 17 have half a glass on a special occasion. I wouldn’t think I’ve done the wrong thing* (P4)

*That’s the only thing but when he had that even 2 years ago, when he was 12, ‘can I have a sip’ I wouldn’t have let him…just this age* [14]*, he’s taller than me, he said ‘can I’ and I went if you want a taste that’s fine but you won’t like it.* (P1)

The perception that this behaviour was appropriate – and even desirable – clearly differentiated them from the ‘bad’ parents who encourage or allow (problematic, unsafe or excessive) underage drinking in their children, and parents who ‘supply’ their children with alcohol.

### Response to previous campaign materials

The perception that ‘underage drinking’ did not include the drinking these parents facilitated among their own children enabled them to understand ‘underage drinking’ and parental ‘supply’ of alcohol as problems only for ‘other’ children in ‘other’ families outside their own social circle. This idea was articulated very clearly in the parents’ responses to the sample underage drinking campaigns.

While we provided a wide range of examples of different messages and approaches (e.g.: legal implications, social norms messages, pledges not to supply), many of the parents were attracted to the high-fear graphic advertising. It was these campaigns they described as being the most impactful and effective for the ‘target audience’. However, this group of parents clearly differentiated themselves and their families from ‘the target audience’, providing a number of suggestions as to how these images could be made more graphic and thus, in their opinion, more effective amongst parents who, unlike themselves, supply alcohol to their children.

*I think it relates back to what we were saying before about those ads on TV where there’s the girl with her pants down and spewing in the gutter and you go ‘oh, I don’t want that to be my child’ and these ones…. I like those* (P1)

*With the one with the boy – I’d probably have a bit more vomit down*

*Something running out of his ear*

*Blood somewhere*

*A little bit of blood wouldn’t go astray*

*Is he in prison there?*

*Yeah*

*There are no bars*

*Put a couple of bars or something* (P2)

*The one that scared the living daylights out of you and put the blame on the mother* (P2)

The same response was evident in the adolescent groups, with the participants offering suggestions for ways that the messages could be made ‘more effective’ for ‘those kids’ who, unlike themselves or their friends, engage in (unsafe, excessive or problematic) underage drinking. Their suggestions also consisted of making the images more graphic.

*This guy looks like he’s about to kill himself*

*Yeah*

*I think this one’s the best* (A1)

*I reckon its good having a colourful thing like that or something really serious, like someone throwing up, something really bad – so people see it and go ‘oh crap’ I don’t want that to happen*. (A2)

*And I would put a picture like that, hurt, or being*

*Really vivid*

*Yeah, like what x said, with death.* (A4)

However, it is important to note that many adolescents recounted stories of events that they had witnessed, or that they had heard about from their peers, that were more shocking than any posters we could have shown them. These recalled outcomes of underage drinking ranged from loss of bladder control to public stabbings. They also reported that these experiences had not deterred them from drinking alcohol.

*Last year, at Carols by Candlelight a guy in your year* (pointing to other participant) *he got drunk that much he peed himself heaps* (A2)

*When teenagers drink they become aggressive and…you know the town hall in Gxxxx?....The other week there was a stabbing at the town hall, it was in the middle of the night. I think a cleaner went past and there was a group of drunk teenagers and they stabbed an adult, like an older person…Yeah. They had no reason to do it; they just came out of a party and stabbed him* (A1)

*You wouldn’t be worried then but there were these people and they were drinking on New Year’s Eve and the fireworks went off and they were drinking and the fireworks finish at 9 so they decided to go to Sxxx Beach and they went to Sxxx Beach and stayed there and then they went home. And they woke up the next morning and realised one of them wasn’t there and then they went looking everywhere and they found him passed out, and they’d left him there and they didn’t even realise* (A4)

*There was a party in Bxxxx and a girl got stabbed in the neck when I was there and it was meant to be just a normal party and she got stabbed in the neck. Yeah, someone as a joke was ‘oh yeah, go to a party in Bxxxx, bring a knife’ as a joke and it fell out of his pocket and these girls had a fight. I saw them have a fight and then they walked away – some chick just picked it up and before you knew it X was just on the ground. And then it was just crazy. And then they were all punching the police cars. Everyone was violent and drunk* (A4)

## Discussion

The Theory of Planned Behaviour (TPB) posits that the predictors of behavioural intention are attitudes, subjective norms, and perceived behavioural control [41, 42]. In the context of parental supply of alcohol, this suggests that if parents believe that supplying alcohol to children and teenagers is wrong (desired attitudes), that the majority of their peers do not provide alcohol to children and teenagers (desired subjective norms) and that the provision of alcohol to children and teenagers is within their control (desired perception of behavioural control), they will not provide alcohol to their children before they reach the age of 18 (the legal alcohol purchase age in Australia).

However, it is clear from the results of this study and from existing national data [7], that many parents *do* provide alcohol to their underage children. Consistent with previous research [20, 53, 54], adolescents in our focus groups were more likely to report that their parents provided them with alcohol than parents (some of whom came from the same families, and all of whom came from the same small community) were to report doing so.

Our participants wholeheartedly agreed with the messages (they thought) we and ‘the government’ were communicating – that supplying alcohol to teenagers is inappropriate. They interpreted ‘supply’ to refer to the provision of quantities of alcohol for unsupervised drinking, such as for consumption at parties, and clearly distanced themselves from people who would engage in this behaviour. The homogeneity of the responses was noteworthy, with all of the parents communicating that they would not ‘supply’ alcohol and almost all providing small amounts or tastes of alcohol in specific contexts. It is possible – given that this behaviour is associated with moral censure and ‘bad’ parenting – that our recruitment strategy did not attract parents who provide larger amounts of alcohol to their children and/or that some of the participants do indeed do so but were unwilling to state this in the group context.

The provision of alcohol to children and adolescents can broadly be described as three behaviours: allowing children to sip or taste alcohol; allowing them to have a drink of alcohol at home; and supplying them with alcohol to take to a party. While each of the behaviours may be potentially harmful, the degree of harm and the consistency of research evidence for harm (or benefit) vary, as do parental and community attitudes. The findings of the present study are consistent with previous evidence that parents see a clear distinction between ‘sipping’ and ‘drinking’; for example, an earlier Australian study which found that parents reported having strict rules prohibiting their children from drinking but allowed them to ‘sip’ or ‘taste’ alcohol [55]. Parents interviewed for this study clearly perceived their own behaviour – providing (small amounts of) alcohol to their teenage children – to be a fundamentally different (and appropriate) behaviour. They were ‘teaching their children to drink’ and their children were learning that their parents would provide them with alcohol for consumption in ‘safe’ places, in ‘safe’ quantities, with their families or with other ‘good’ children. These parents do not perceive any correlation between their behaviour and excessive, problematic or unsafe alcohol use and they thus do not identify with the characters or scenarios depicted in recent fear-based social marketing campaigns – which they see as targeted at problematic ‘other’ parents and children. As a result, they remain uninfluenced by campaigns targeting parental supply.

In turn, many of the (older) adolescents in our focus groups perceive that their parents condone or even encourage them to consume alcohol prior to reaching 18 years of age and, like their parents, do not perceive that underage drinking campaigns are targeted at people like them. It appears these children had learned from their parents that supplying alcohol does not include allowing children to taste or sip alcoholic drinks at home and that underage drinking does not include drinking that takes place in the presence, or with the permission, of parents.

The findings of this study have important implications for the development of communication materials and social marketing campaigns targeting underage drinking, and particularly parental supply. It is essential to ensure that the target audience perceive themselves to be the target audience, and are not in fact commenting on the effectiveness of the message for ‘other’ people. The frame of reference for, and interpretation of terms by, target audiences can be fundamentally different to that of message developers. High-fear graphic images may test well with adolescents and parents because they genuinely believe these are the most effective strategy for ‘those’ children and ‘those’ parents. Following their recommendations is, then, likely to result in campaigns that the intended target audience will not perceive as relevant to them, or as addressing their own behaviour. These messages will not impact on their attitudes or behaviours because they believe they are already doing the ‘right’ thing.

Thus, the first goal of such campaigns should be to ensure that the target audience perceives themselves to be the target audience; which can be achieved by utilising images and words that ‘nice’ parents with ‘good’ children identify with. For example, in our subsequent development of messages for this community, rather than utilising stock images that are reminiscent of previous high fear advertising campaigns we are taking photographs of clean-cut children in recognisable local environments. We are also using the words of our focus group participants in messages and taglines; for example, as one parent stated when she realised that her own children were also at risk of harm: “Bad things happen to good kids too”.

The findings also have implications for addressing the entrenched behaviour of parental supply of alcohol to teenagers by their parents (which is both legal and socially accepted in Australia). Strategies to reduce parental supply, and thus underage drinking, need to increase parents’ awareness of the negative short- and long-term effects of any actions that condone underage drinking in any context, including providing small amounts of alcohol to children and teenagers in private homes.

### Limitations

This study was conducted in one regional town in eastern Australia, and thus the results may not be generalizable to larger cities or other regions; future research in other locations could explore similarities and differences in perspectives. The use of written materials (in English) for recruitment limited our ability to attract participants who were not fluent in English, had lower levels of literacy, or were otherwise hard-to-reach groups. While the use of Facebook as an additional recruitment strategy broadened our reach, we note that all of our participants were fluent English speakers, with 93 % speaking English at home, and all were sufficiently literate to read the participant information sheet and provided informed consent. We utilised mixed-gender focus groups to explore these behaviours as young people typically drink in mixed-gender social groups and parents make decisions about supply with their spouses. However, the use of mixed-gender focus groups meant that we did not explore gender differences in the participants’ responses. Future research could usefully explore differences between male and female adolescents (and between mothers and fathers) in both perceptions of the risks associated with underage drinking and the most salient social marketing messages.

## Conclusions

Communication materials and social marketing campaigns targeting underage drinking and parental supply need to be carefully developed and tested with the target audiences to ensure that the message developers and the audience have the same understanding of the behaviour that is being targeted. It is also important to ensure that the target audiences recognise themselves as such, and do not interpret the messages as important and effective for ‘other’ people.

## Abbreviations

LGA, local government area; NSW, New South Wales; SEIFA, socio economic index for area; TPB, theory of planned behaviour

## Footnotes

1 Data were not reported separately for supply by own parents vs friends’ parents.

2 The SEIFA is standardised against a mean of 1000 with a standard deviation of 100; the middle two-thirds of SEIFA scores fall between 900 and 1100.
